# Acute bilateral optic/chiasm neuritis with longitudinal extensive transverse myelitis in longstanding stable multiple sclerosis following vector-based vaccination against the SARS-CoV-2

**DOI:** 10.1007/s00415-021-10647-x

**Published:** 2021-06-15

**Authors:** Christoph Helmchen, Gesine M. Buttler, Robert Markewitz, Katja Hummel, Heinz Wiendl, Tobias Boppel

**Affiliations:** 1 Department of Neurology, University of Lübeck, University Hospitals Schleswig Holstein, Campus Lübeck, Ratzeburger Allee 160, 23538 Lübeck, Germany; 2 Institute of Clinical Chemistry, University Hospitals Schleswig-Holstein, Campus Lübeck, Kiel, Germany; 3 Department of Neurology with Institute of Translational Neurology, University Hospital Münster, Münster, Germany; 4 Institute of Neuroradiology, University Hospitals Schleswig-Holstein, Campus Lübeck, Lübeck, Germany

Dear Sirs,

Multiple sclerosis (MS) and neuromyelitis optica (NMO) are two distinctly different immunological diseases with respect to clinical and MRI signs [4, 10], clinical course, therapy, and pathoimmunology [e.g., antibodies targeting astrocytic water channel aquaporin-4 (AQP4-ab), redefining the variety of clinical NMO as NMO spectrum disorder (NMOSD)] [10, 11]. Diagnostic criteria are reliable so that both entities should not overlap and must not be confused, e.g., AQP4-ab were only found in 0.33% of 1183 patients with longstanding MS [3].

We report about a young patient with a longstanding history of relapsing-remittent multiple sclerosis (RRMS) who developed a severe syndrome of optic/chiasm neuritis and paraplegia due to longitudinal extensive transverse myelitis (LETM) [6] resembling NMOSD 2 weeks after her vaccination with the first dose of a vector-based COVID-19 vaccine.

The 40-year-old female patient has a longstanding (21 years) history of RRMS. Diagnosis of RRMS was established in 2000 based on relapsing neurological episodes of different symptoms with variable lesion location (diplopia, paresthesia, paresis of right hand, but no signs of optic neuritis), typical cervical and brain MRI lesions suggestive for RRMS, and intrathecal oligoclonal bands. Other causes were excluded.

In the first few years, she was treated with interferon and glatiramer acetate and steroid infusions. As she had developed cervical myelitis (at the level C4/5) and continued to suffer from several annual relapses (3–4 x/year), she was put on Natalizumab (NAT) in 2009. Ever since she has had only a few and mild episodes of sensory symptoms but remained functionally independent in daily life. She was repetitively tested negative for antibodies against NAT and JC virus.

**Fig. 1:**
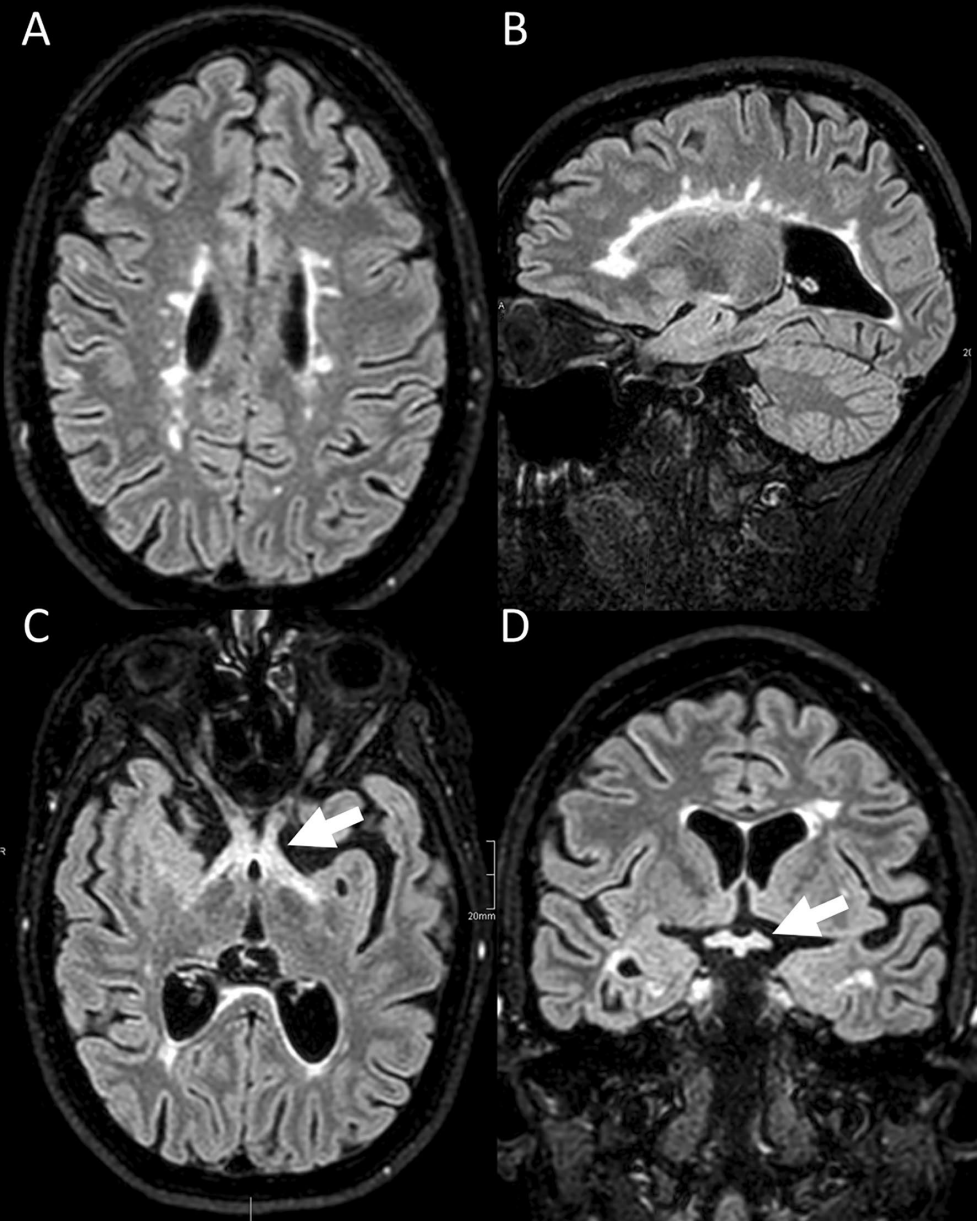
Cranial MRI (1.5T, FLAIR images) shows an acute inflammatory chiasm lesion (suggestive for NMOSD) in this patient with typical MS lesions. There were numerous chronic periventricular Dawson finger-shaped and ovoid demyelination lesions on axial (**A**) and sagittal (**B**) fluid-attenuated inversion recovery (FLAIR) images compatible with MS and an increased signal intensity in the chiasm (white arrow) and part of the adjacent optic nerves and optic tract on axial (**C**) and coronal (**D,** white arrow) images

**Fig. 2:**
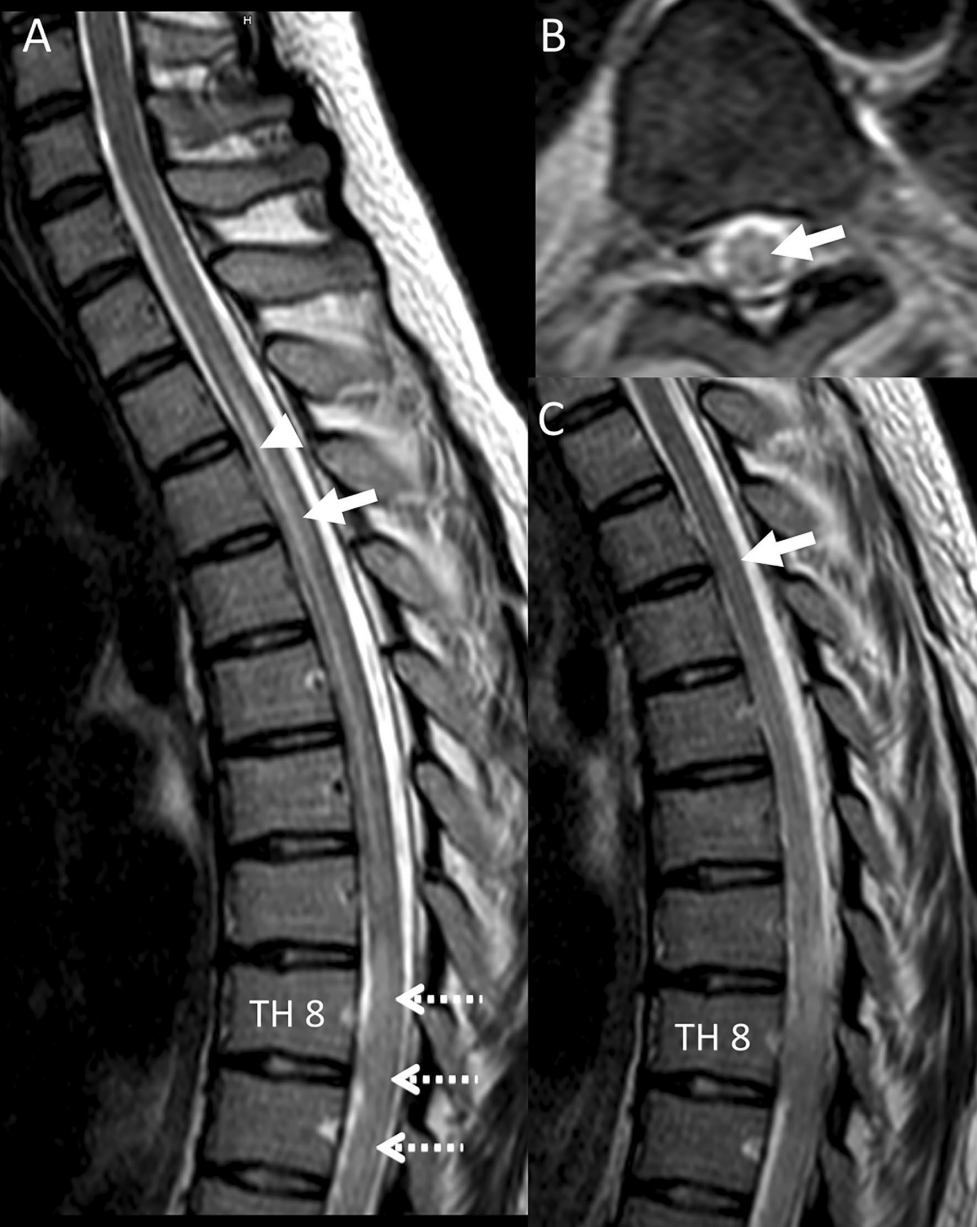
Spinal MRI shows longitudinal thoracic myelitis. Spinal MRI (1.5T, T2w) revealed longitudinal centrally located T2w signal intensities throughout the thoracic myelon (**A**, lucencies marked by white arrow on sagittal images) indicating an extensive thoracic myelitis with maximal extent at TH7-10 on axial (**B**, 1 week later) and sagittal (**C**) T2w images. In the acute stage (**A**), the central canal (white arrowhead) was slightly enlarged in the thoracic myelon (**A**) above the maximal swelling at Th7–10 (white dashed arrows) which slightly reversed on follow-up MRI (1 week later; **B**, **C**). However, severe longitudinal myelitis persisted (**C**); note the centrally located inflammation of the thoracic myelon on the axial slice (**B**)

Based on the stable disease without severe relapses, she received a vector-based vaccination against SARS-CoV-2 in March 2021 (Astra Zeneca, COVID19 Vaccine^®^; Vaxzevria^®^), 8 days after the last NAT infusion, and had no immediate adverse events. Two weeks after the vaccination, she noticed blurring of vision which rapidly developed to binocular blindness within 48 h. On day 2 of her blindness, she noticed back pain, mild weakness and numbness in her legs which escalated to the inability to walk within the next 24 h. On day 3, she was functionally blind and the paraparesis deteriorated to paraplegia, with absent tendon reflexes in the legs, incontinence, and a sensory deficit for all qualities below Th5. CSF showed severe pleocytosis (524 leucocytes/µl, 98% neutrophil granulocytes), increased lactate (6.6 mmol/l) and strongly elevated protein (2.2 g/l). Cranial MRI revealed numerous old white matter lesions compatible with MS (in the corpus callosum and periventricular white matter; Fig. 1A, B) and increased signal intensity in the chiasm and part of the adjacent optic nerves and tracts (Fig. 1C, D), MR-morphologically in line with NMOSD. Only mild contrast enhancement of the optic chiasma was observed. Note that there had not been optic nerve/chiasm involvement in previous cranial MRI. Visual-evoked cortical potentials could not be elicited bilaterally. Spinal MRI (1.5T) revealed increased longitudinal centrally located signal intensities throughout the thoracic myelon indicating a myelitis with maximal extent at TH7-10 (Fig. 2A) and in the medullary conus. Mildly elevated signal intensities were also seen at C7-Th1. There were additional residual post-myelitic lesions in the cervical spine (level C4/C5). Two days after having had received methylprednisolone (2 g/day), there was no contrast enhancement visible. Initially, there was a slight enlargement of the central canal of the thoracic myelon above the maximal swelling at Th7-10 (Fig. 2A), which subsided in a follow-up MRI 1 week later (Fig. 2B, C). A broad autoantibody panel was negative including those targeting myelin oligodendrocyte glycoprotein [MOG, assessed via life-cell-assay [2] and confirmed via indirect immuno-fluorescence testing with MOG-transfected HEK-293 cells in another reference lab (EUROIMMUN, Lübeck, Germany)], glial fibrillary acid protein (GFAP), flotillin and AQP4 (in serum and CSF). She was tested negative for acute responses to several virus antigens (herpes simplex HSV1/2, varicella zoster virus, Epstein–Barr, German measles, mumps, and rubella) and bacteria (borrelia burgdorferi, mycoplasmas, and syphilis). Serological testing for antinuclear and anti-phospholipids antibodies were negative. Repetitive PCR tests for SARS-CoV-2 were negative. We refrained from SARS-CoV-2 antibody testing after prolonged plasmapheresis but there were no antibodies during sequential testing in 2020 until 2 weeks before symptom onset. The MRZ reaction was negative at the last lumbar punction.

**Fig. 3:**
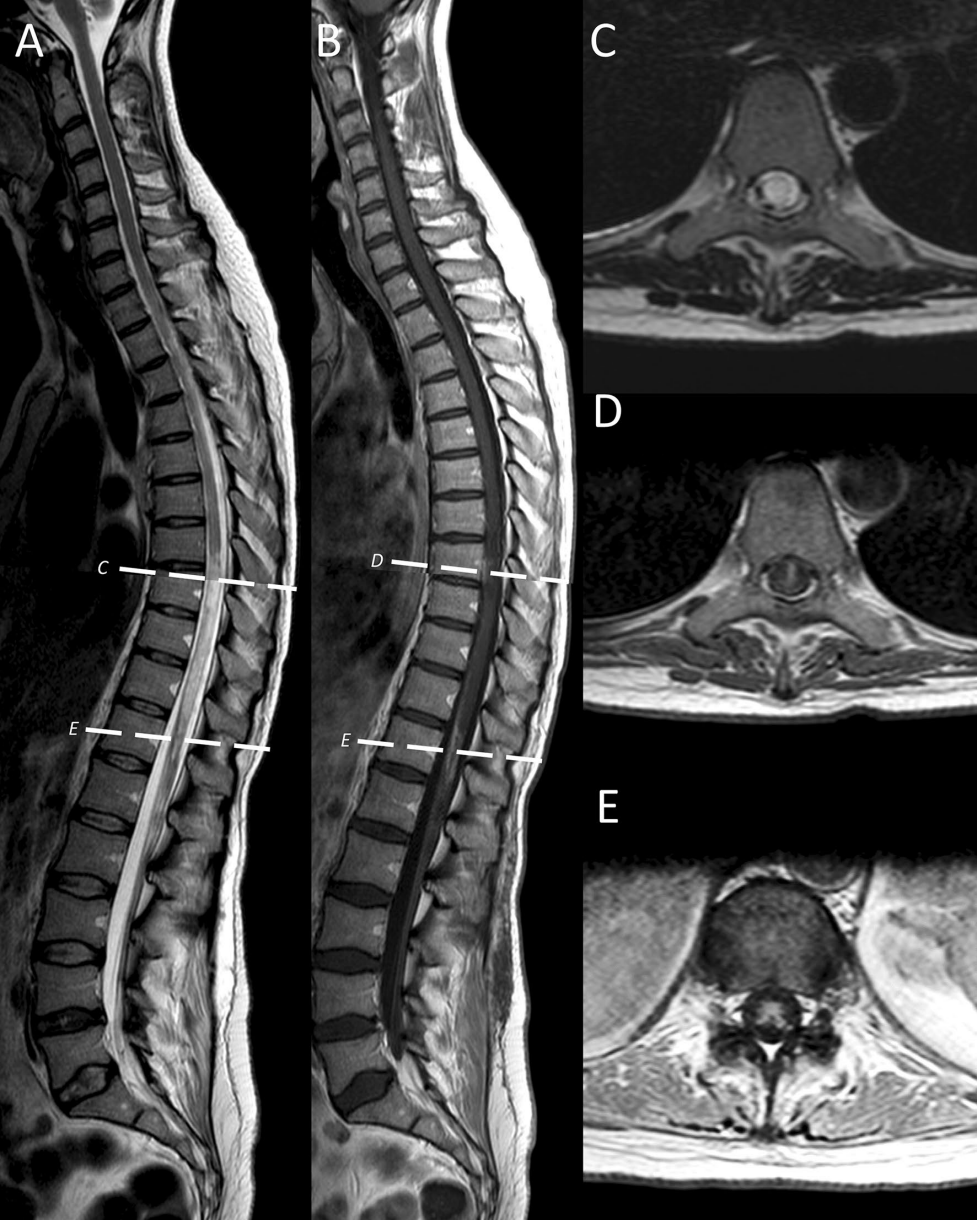
Two months after subacute onset follow-up spinal MRI (no steroid therapy) shows severe chronic LETM with still some active inflammation at the same sites. From left to right: compared to the images at subacute onset (Fig. 2; with steroids) sagittal spinal MRI (1.5T, T2w) revealed much stronger and more extensive longitudinal T2w signal intensities (**A**) throughout the thoracic myelon from Th7 to the conus, and in (**B**) stronger enhancement in the conus and at TH8 in the T1w images with contrast. The transverse slices on the right show the corresponding slices at the level of TH8 and the conus indicated by the white hatched lines in the sagittal images. **C** Transverse T2w images; **D**, **E** transverse T1w images with contrast at the levels indicated in the sagittal slices

In addition to steroids, she was treated with plasmapheresis and immunoadsorption with slight recovery of visual functions (recognition of motion but no objects) but paraplegia, loss of sensory function below T5, and incontinence persisted. Weekly follow-up examinations in CSF showed improved pleocytosis (33/µl; 48/µl with a switch to lymphocytic pleocytosis) but even further increased protein (up 4.9 g/l). NAT was continued. Two months after subacute onset, with even more improved visual acuity but unchanged paraplegia follow-up spinal MRI still revealed even stronger contrast enhancement in the thoracic myelon (at TH7-8) and in the conus, in the absence of steroids (Fig. 3). Moreover, the longitudinal T2w signal intensities in the thoracic myelon down to the conus became more intense and homogenous (Fig. 3). Cranial MRI did not show contrast enhancement and the signal intensities in the chiasm almost disappeared. CSF, however, improved and did not show lymphocytic pleocytosis any longer (4/µl) with still elevated but improved protein (1 g/l).

Although potentially co-incidental, we have to consider the vector-based anti-SARS-CoV-2 vaccine as a main cause or at least a trigger of the severe clinical course in this RRMS patient for the following reasons:

The onset of symptoms 2 weeks after the vaccination is in line with a dysimmunological response phenotypically presenting with optic/chiasm neuritis and LETM resembling NMOSD. There was no evidence for a previously unrecognized NMOSD [3]. Transverse myelitis (TM) and LETM have been described in patients suffering from COVID-19 [7] but repetitive tests for SARS-CoV-2 were negative in our patient. A recent meta-analysis of adverse events across different vaccines against COVID-19 revealed a very low risk (1/10,000) of TM in the general population [5]. TM has not been reported in MS patients treated with BNT162b2 vaccine (Pfizer-BioNTech COVID-19) injections [1] but there were 3 patients in the safety register of the approval study of the vector-based COVID-19 Vaccine^®^ [7, 9]. One of them turned out to suffer from a previously unrecognized MS.

The nearly concurrent manifestation of chiasm and longitudinal myelitis argues against a relapse of RRMS. It may be speculated that the intended B cell response targeting the vector-based anti-SARS-CoV-2 vaccine triggered a dysimmunological process with a possibly pre-existing B cell pathoimmunology, with an ADEM-like excessive activity. In antibody-driven NMOSD, peripheral plasma cells are the source for AQP4-ab crossing the disrupted blood–brain barrier targeting astrocytes. NAT usually leads to activation and accumulation of B cells in the peripheral blood but prevents immune cells from crossing the (intact) blood–brain barrier. NAT could have induced catastrophic disease activity in this AQP4-ab negative NMOSD-like syndrome [8]. However, as this had not occurred during 10 years of regular NAT treatment, NAT is unlikely to be the cause for the acute LETM.

Our case suggests that the vector-based COVID-19 vaccine should not be used in RRMS if mRNA vaccines are available.
